# The multifaceted roles of PP2C phosphatases in plant growth, signaling, and responses to abiotic and biotic stresses

**DOI:** 10.1016/j.xplc.2025.101457

**Published:** 2025-07-12

**Authors:** Hossein Ghanizadeh, Zainab Qamer, Yao Zhang, Aoxue Wang

**Affiliations:** 1 College of Horticulture and Landscape Architecture, Northeast Agricultural University, Harbin 150030, China; 2 College of Life Sciences, Northeast Agricultural University, Harbin 150030, China

**Keywords:** phosphorylation, ABA signaling, MAPK cascade, plant immunity, genetic regulation, crop improvement

## Abstract

Abiotic and biotic stresses constitute substantial challenges to global agricultural productivity. Plants have evolved complex regulatory mechanisms to mitigate these stresses, including signaling networks that govern physiological, biochemical, and molecular responses. Among these, protein phosphorylation plays a pivotal role in stress adaptation; protein phosphatase 2C (PP2C) proteins serve as key regulators across multiple signaling pathways. PP2Cs influence plant stress responses through interactions with various proteins, including SNF1-RELATED PROTEIN KINASE 2s (SnRK2s), abscisic acid (ABA) receptors, transcription factors, and ion channels, thereby fine-tuning signaling cascades and physiological adaptations under stress conditions. This review provides a comprehensive analysis of the PP2C gene family in plants, emphasizing their structural characteristics, regulatory mechanisms, and functional roles in abiotic and biotic stress responses. We highlight the biological significance of PP2Cs in modulating critical pathways such as ABA, mitogen-activated protein kinase (MAPK), and calcium signaling. Additionally, we explore the roles of PP2Cs in regulating root development, stomatal behavior, ion homeostasis, and immune responses, demonstrating their roles in coordinating plant developmental processes with adaptive responses to environmental challenges, particularly under resource-limited conditions. Finally, we identify current knowledge gaps and propose future research directions to advance the broader understanding of PP2C-mediated regulation of physiological functions, stress responses, and developmental signaling. Deeper insights into PP2C functions may facilitate novel strategies to promote crop resilience and enhance agricultural sustainability.

## Teaser

Protein phosphatase 2C (PP2C) proteins serve as key regulators in plant growth, signaling, and responses to abiotic and biotic stresses. This review provides a comprehensive overview of the PP2C gene family in plants, with a focus on their regulatory mechanisms and functional roles in stress responses. It highlights the importance of PP2Cs in modulating critical pathways such as ABA, MAPK, and calcium signaling, and explores their involvement in integrating developmental processes with adaptive responses to environmental challenges. Advancing our understanding of PP2C functions may inform strategies to enhance plant resilience and improve agricultural sustainability.

## Introduction

Abiotic and biotic stresses pose major threats to sustainable agricultural production worldwide, limiting the ability to meet the food demands of a growing global population (Hossain et al., 2021). Under stress conditions, crops often experience a range of physiological disturbances, including osmotic and oxidative stress, impaired photosynthesis and metabolism, and nutrient imbalances, all of which can reduce productivity. To counter these adverse effects, crops have evolved complex regulatory systems that engage signaling networks to coordinate physiological, biochemical, and molecular responses (Hossain et al., 2021).

Protein phosphorylation is a central signaling mechanism regulated by protein kinases (PKs) and protein phosphatases (PPs), which together control numerous cellular functions, including environmental stress responses, growth signaling, hormonal pathways, metabolic activity, and development (Chen et al., 2021). PKs catalyze the phosphorylation of serine (Ser), threonine (Thr), and tyrosine (Tyr) residues, whereas PPs remove phosphate groups to reverse this modification (Yang et al., 2022). According to substrate specificity, PPs are classified into three major groups: protein tyrosine phosphatases, serine/threonine phosphatases, and dual-specificity phosphatases (Ruan et al., 2024). The serine/threonine phosphatases are further divided into two gene families—protein phosphatase P (PPP) and protein phosphatase M (PPM)—based on differences in amino acid sequences and crystal structures. The PPP family includes key phosphatases such as PP1, PP2A, and PP2B; the PPM family includes PP2C and pyruvate dehydrogenase phosphatase (Rodriguez, 1998). PP2Cs regulate kinase signaling cascades across all domains of life (Archaea, Bacteria, and Eukarya). Within the Eukarya domain, they function in multiple kingdoms, including Viridiplantae (land plants) (Schweighofer et al., 2004). In vascular plants, PP2C proteins regulate signaling pathways through multiple mechanisms, including modulation of kinase activity, ion channel regulation, and control of transcriptional regulators (Chuong et al., 2020). This review examines the biochemical and molecular basis of the plant PP2C phosphatase family, providing an overview of their roles in modulating diverse biological functions in plants.

## Structural and regulatory features of PP2Cs

### Structures of PP2Cs

PP2Cs form a highly conserved family of serine/threonine phosphatases with essential roles in regulating plant stress signaling and development (Bhaskara et al., 2019). All PP2Cs possess a conserved catalytic core of approximately 300 amino acids, comprising 11 invariant motifs required for enzymatic function (Schweighofer et al., 2004). Although they exhibit low sequence homology with PPP-type phosphatases, their three-dimensional structures are strikingly similar, indicating evolutionary convergence on a common catalytic strategy (Lammers and Lavi, 2007). In most *Arabidopsis thaliana* PP2Cs, the catalytic domain is located at the C-terminus, although N-terminal placement also occurs (Chuong et al., 2020). For instance, in clade B PP2Cs, the catalytic core is directly connected to an N-terminal region containing a kinase-interacting motif (Schweighofer et al., 2004). Structural studies have highlighted the importance of surface loops and specific motifs within the catalytic core in mediating protein–protein interactions (Seok, 2021). High-resolution analyses have shown that PP2Cs use flexible surface loops to bind interaction partners in a complex-specific manner (Soon et al., 2012). In HAB1, a clade A PP2C, a hydrophobic “flap” and adjacent tryptophan “lock” insert into the gate-and-latch loops of the abscisic acid (ABA) receptor, stabilizing the phosphatase–receptor complex (Melcher et al., 2009). These observations emphasize that loop-based docking determines PP2C specificity, enabling functional versatility across diverse plant signaling networks.

### Regulatory mechanisms of PP2Cs

Regulation of PP2C activity in plants occurs through multiple modulatory layers beyond transcriptional control. At the post-translational level, proteolysis and ubiquitin-mediated degradation serve as key mechanisms to limit PP2C abundance and enable precise temporal regulation. For example, clade A PP2Cs, such as ABI1, are ubiquitinated by E3 ligases PUB12 and PUB13 in an ABA-dependent manner, targeting them for degradation and thus facilitating stress signal transduction (Kong et al., 2015; Pan et al., 2020). Subcellular localization also restricts the functional reach of PP2Cs by modulating substrate access (Bhaskara et al., 2019). Members of clade D PP2Cs, including PP2CD1, PP2CD3, and PP2CD4, are found in both the nucleus and cytosol, whereas PP2CD8 localizes predominantly to mitochondria (Tovar-Mendez et al., 2014). Organelle-specific localization, as observed in clade D members, ensures spatial coordination of PP2C activity with upstream kinases and downstream effectors. Additionally, PP2C activity can be regulated by sequestration into inactive complexes. For instance, ROP11, a plant-specific Rho-like small GTPase, suppresses ABA signaling by binding ABI1 at the plasma membrane (PM). This interaction shields ABI1 from PYRABACTIN RESISTANCE 1 (PYR1)/PYR1-LIKE (PYL) receptors, maintaining it in a functionally inert complex (Li et al., 2012). Allosteric modulation also contributes to PP2C regulation. The adaptor protein EAR1 (Enhanced ABA Repression 1) acts as an allosteric activator of clade A PP2Cs, substantially enhancing their catalytic activity (Wang et al., 2018). Moreover, some PP2Cs are subject to additional regulatory influences, including redox modification, phosphorylation, and interactions with non-coding RNAs (Li et al., 2021; Ai et al., 2022; Bi et al., 2022). These diverse mechanisms extend PP2C regulation beyond transcriptional and proteolytic control.

Overall, the structural integrity and domain organization of PP2Cs provide the biochemical basis for their regulatory flexibility. This versatility is further amplified by multilayered controls at transcriptional, post-translational, and spatial levels, positioning PP2Cs as central integrators of plant stress and developmental signaling.

## Biological functions of PP2Cs

### Roles of PP2Cs in hormonal signaling

#### ABA signaling

**Figure 1:**
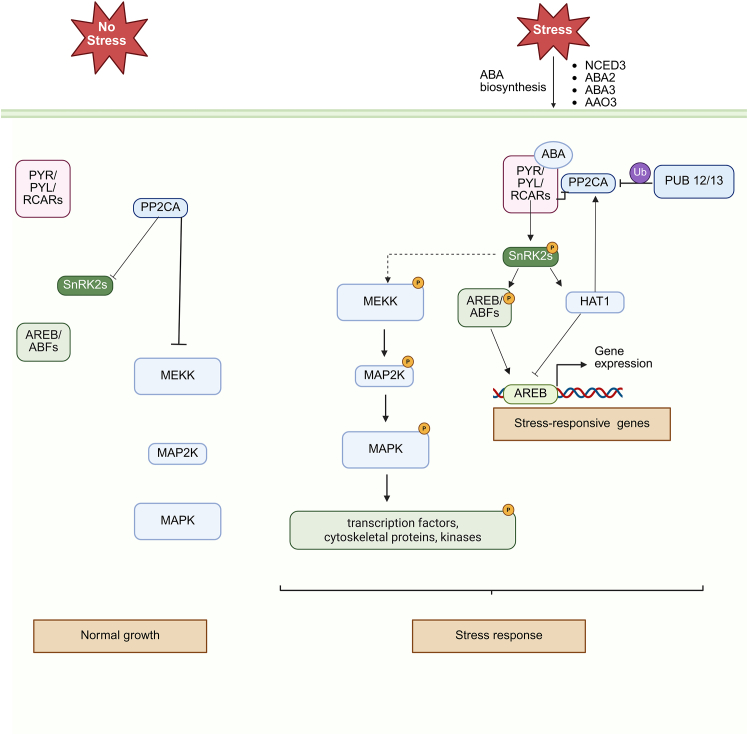
ABA-mediated regulatory network during plant stress responses. Under non-stress conditions, clade A protein phosphatase 2Cs (PP2CAs)—including ABA-INSENSITIVE 1 (ABI1), ABI2, and HYPERSENSITIVE TO ABA1 (HAB1)—repress abscisic acid (ABA) signaling by dephosphorylating and inhibiting SNF1-RELATED PROTEIN KINASE 2s (SnRK2s). This prevents activation of downstream components such as the mitogen-activated protein kinase (MAPK) cascade, comprising MAPK kinase kinase (MEKK), MAPK kinase (MAP2K), and MAPKs, as well as ABA-responsive transcription factors including ABA-RESPONSIVE ELEMENT-BINDING proteins (AREBs) and ABRE-BINDING FACTORS (ABFs). In response to abiotic stress, ABA is synthesized through the activity of enzymes such as 9-*cis*-epoxycarotenoid dioxygenase 3 (NCED3), ABA2, ABA3, and aldehyde oxidase 3 (AAO3). ABA binds to its receptors—PYRABACTIN RESISTANCE 1 (PYR1), PYR1-LIKE (PYLs), and REGULATORY COMPONENTS OF ABA RECEPTORS (RCARs)—forming a complex that inhibits PP2CAs, thereby releasing SnRK2s from repression. Activated SnRK2s phosphorylate AREB/ABF transcription factors, inducing the expression of stress-responsive genes through ABA-responsive elements (ABREs). SnRK2s also activate the MAPK cascade, which regulates transcription factors, cytoskeletal proteins, and kinases involved in stress adaptation. Additionally, SnRK2s phosphorylate the HD-ZIP transcription factor ARABIDOPSIS THALIANA HOMEOBOX PROTEIN 1 (HAT1), which suppresses ABA biosynthesis and ABA-responsive gene expression while promoting transcription of PP2CA genes, establishing a negative feedback loop to modulate ABA signaling. Furthermore, ABA-bound PP2CAs are targeted for degradation via ubiquitination by E3 ubiquitin ligases such as PLANT U-BOX 12 and 13 (PUB12/13), contributing to signal fine-tuning. Sharp arrows and blunt arrows indicate promotive and inhibitory effects, respectively. Created with www.Biorender.com.

*Clade A members.* Clade A protein phosphatase 2Cs (PP2CAs) serve as key negative regulators of ABA signaling. This group includes ABI1, ABI2, AHG1, AHG3 (also known as AtPP2CA), HAB1, HAB2, HAI1, HAI2 (PP2C1), and HAI3 (Schweighofer et al., 2004). Whereas several PP2CAs, such as ABI1 and ABI2, act through the core ABA signaling pathway, others, including HAI1, HAI2, and HAI3, contribute to ABA-independent stress responses (Bhaskara et al., 2012). Extensive research has characterized the roles of PP2CAs in ABA signaling (Figure 1). In the absence of ABA, PP2CAs interact with and dephosphorylate SNF1-RELATED PROTEIN KINASE 2s (SnRK2s), particularly at Ser176 within their activation loops, thereby maintaining these kinases in an inactive state and suppressing ABA-responsive gene expression (Jossier et al., 2009). Structural studies have shown that a conserved tryptophan residue in PP2CAs aligns within the catalytic cleft of SnRK2s, stabilizing the inactive complex (Yunta et al., 2011). Upon perception of ABA, PYR1, PYLs, and REGULATORY COMPONENTS OF ABA RECEPTORS (RCARs) bind ABA and subsequently associate with PP2CAs. This interaction inhibits PP2CA phosphatase activity, allowing SnRK2 activation (Umezawa et al., 2009). The conserved tryptophan residue in PP2CAs is critical for docking into the receptor complex; mutations near this residue impair PP2CA–PYL interactions, resulting in ABA-insensitive phenotypes (Santiago et al., 2009; Fujioka et al., 2019). Activated SnRK2s phosphorylate downstream effectors, including transcription factors and ion channels, thus promoting ABA-responsive signaling under stress conditions (Fujii and Zhu, 2009).

ABI1 and ABI2 were the first PP2CAs identified as regulators of ABA signaling. Both phosphatases dephosphorylate SnRK2s—particularly SnRK2.2, SnRK2.3, and open stomata 1 (OST1; also known as SnRK2.6)—which serve as key positive regulators in the ABA signaling pathway. HAB1, a close homolog of ABI1 and ABI2 (Rodriguez, 1998), also inhibits OST1 activity. Overexpression of HAB1 induces ABA insensitivity in seeds and vegetative tissues, suppresses ABA-inducible gene expression, and disrupts stomatal regulation (Saez et al., 2004). HAB1 also interacts with the SWI/SNF chromatin remodeling complex via the SWI3B subunit. Loss of SWI3B function increases ABA sensitivity, indicating a positive regulatory role for SWI3B in ABA signaling (Archacki et al., 2016). AHG1 and AHG3, two additional clade A PP2CAs, regulate seed germination through ABA-dependent mechanisms. Both *ahg1* and *ahg3* mutants display hypersensitivity to ABA; AHG3 is inhibited by PYL/RCARs, whereas AHG1 functions independently of this inhibition (Nishimura et al., 2004; Yoshida et al., 2006; Antoni et al., 2011). AHG1 interacts with DELAY OF GERMINATION1, forming a parallel dormancy control module that integrates ABA-independent cues into seed dormancy regulation (Nishimura et al., 2018). HAI-type PP2CAs (HAI1, HAI2, and HAI3) functionally diverge from other clade A members. Although classified within the same clade, they primarily participate in ABA-independent stress responses and selectively interact with a limited subset of ABA receptors, such as PYL5 and PYL7–10 (Bhaskara et al., 2012). Their expression is upregulated during stress conditions, whereas PYL receptor levels decline, suggesting a distinct mode of regulation. Notably, *hai* loss-of-function mutants exhibit ABA-insensitive phenotypes, highlighting the unique regulatory behavior of these PP2CAs (Bhaskara et al., 2012; Zhang et al., 2013).

*Non–clade A PP2Cs in ABA signaling.* Although clade A PP2Cs are the primary negative regulators of ABA signaling, emerging evidence indicates that PP2Cs from other clades also contribute to ABA-related pathways. For example, clade E growth-regulating phosphatases EGR1 (AT3G05640) and EGR2 (AT3G27930) have recently been implicated in modulating ABA signaling. EGR2 and EGR1 attenuate ABA responses by directly targeting SnRK2.2, dephosphorylating a conserved serine residue (Ser31) in the G-loop of its ATP-binding pocket. This regulatory mechanism suppresses ABA signal transduction upstream of transcriptional outputs, representing a distinct mode of modulation (Ding et al., 2019; Li et al., 2024). Clade G PP2Cs have also emerged as components of the ABA signaling network. In *A. thaliana*, AtPP2CG1 (At2g33700) is induced by ABA treatment, and its expression is significantly reduced in ABA-deficient *abi2-3* mutants relative to wild-type plants, suggesting ABA-dependent regulation (Liu et al., 2012). These findings imply that AtPP2CG1 acts downstream of core ABA signaling components and is positively regulated by ABA. In contrast, another clade G member, PP2C49 (At3g62260), influences ABA-responsive gene expression and appears to function through a negative feedback mechanism within the ABA signaling network, although its direct molecular targets remain unidentified (Chu et al., 2021). A clade I phosphatase, TaPP2C158, was recently shown to negatively regulate ABA signaling in *Triticum aestivum* (Wang et al., 2023a). Although its precise function remains unclear, current evidence indicates that TaPP2C158 does not interact with TaPYL4 and does not dephosphorylate TaSnRK2.10 (Wang et al., 2023a). However, it does dephosphorylate TaSnRK1.1, suggesting that it operates outside the core ABA signaling cascade through an alternative branch involving TaSnRK1.1.

Collectively, these findings highlight that ABA signaling is modulated not only by clade A PP2Cs but also by members of clades E, G, and I. These non–clade A phosphatases likely provide tissue-specific or developmentally regulated control over ABA responses through both canonical and noncanonical pathways. Further studies focused on their structural characteristics, spatiotemporal expression patterns, and clade interacting partners will be essential to fully elucidate their roles in ABA signaling.

#### Auxin signaling

**Figure 2:**
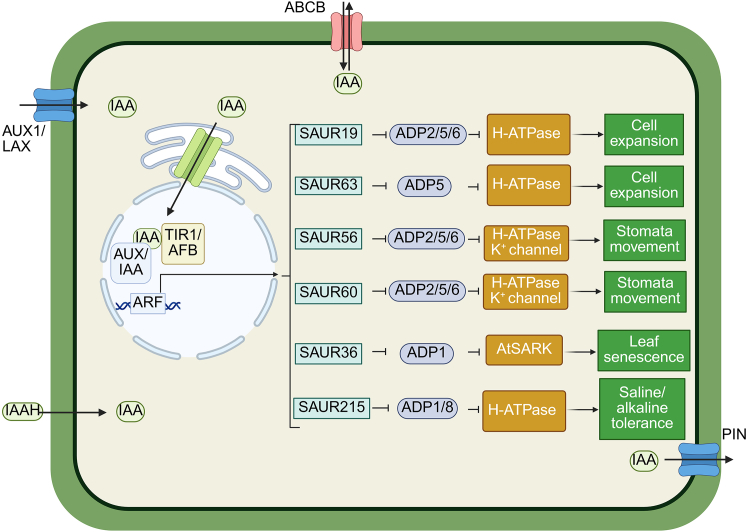
Schematic representation of auxin signaling and its regulation of physiological responses via SAUR–PP2CD modules. Auxin (indole-3-acetic acid [IAA]) enters the cell via AUX1/LAX influx carriers or in its protonated form (IAAH), and is exported by PIN-FORMED (PIN) and ATP-binding cassette subfamily B (ABCB) transporters. Once inside the cell, IAA binds to the TIR1/AFB auxin receptor complex, leading to degradation of AUX/IAA transcriptional repressors and activation of AUXIN RESPONSE FACTORs (ARFs), which induce the expression of auxin-responsive genes, including small auxin members of the SMALL AUXIN UP RNA (SAUR) family. Specific SAUR proteins interact with clade D PP2Cs (ADP proteins) to inhibit their phosphatase activity, thereby activating downstream effectors. SAUR19 and SAUR63 inhibit ADP2/5/6 and ADP5, respectively, promoting plasma membrane (PM) H^+^-ATPase activity and enhancing cell expansion. SAUR56 and SAUR60 suppress ADP2/5/6 to stimulate both H^+^-ATPase and K^+^ channel activity, facilitating stomatal opening. SAUR36 interacts with ADP1 and regulates the SENESCENCE-ASSOCIATED RECEPTOR-LIKE KINASE (AtSARK), contributing to leaf senescence. SAUR215 inhibits ADP1 and ADP8 to enhance PM H^+^-ATPase activity, improving tolerance to saline and alkaline stress. Sharp arrows and blunt arrows indicate promotive and inhibitory effects, respectively. Created with www.Biorender.com.

Within the PP2C family, clade D members play key roles in modulating auxin signaling (Figure 2). Several clade D phosphatases, including ADP2, ADP5, and ADP6, act as negative regulators of SAUR (SMALL AUXIN UP RNA)-mediated pathways (Spartz et al., 2014; Ren et al., 2018). These clade phosphatases possess a conserved C-terminal motif that is essential for interaction with auxin-induced SAUR proteins (Huang et al., 2023a). SAUR genes are rapidly induced by auxin and regulate processes such as activation of plasma membrane (PM) H^+^-ATPases. Specifically, SAUR19 and SAUR63 promote phosphorylation of PM H^+^-ATPases (e.g., AHA1), enhancing their activity (Spartz et al., 2012, 2014). ADP2, ADP5, and ADP6 antagonize this process by dephosphorylating the penultimate C-terminal threonine residue (Thr^947^), thus suppressing ATPase activity (Spartz et al., 2012). This repression is counteracted by SAUR19 and SAUR63, which inhibit clade D phosphatase activity, allowing phosphorylation to persist (Nagpal et al., 2022). These clade D phosphatases also influence stomatal aperture by dephosphorylating PM H^+^-ATPases and associated potassium channels. SAUR56 and SAUR60 promote stomatal opening by suppressing clade D PP2C activity and enhancing the function of these ion channels (Wong et al., 2020). Other clade D members, such as ADP1 and ADP8, also interact with PM H^+^-ATPases, including TaHA2, by dephosphorylating regulatory residues (e.g., Thr^926^; Cui et al., 2023), although their interactions with SAUR proteins have not been confirmed. Given that only a subset of SAURs has been experimentally shown to antagonize PP2CDs, and given the structural and functional diversity of the SAUR family, it is plausible that additional SAUR proteins—particularly those with conserved motifs or membrane localization—also regulate PP2CD activity.

### Roles of PP2Cs in stomatal development and movement

#### Stomatal lineage regulation

**Figure 3:**
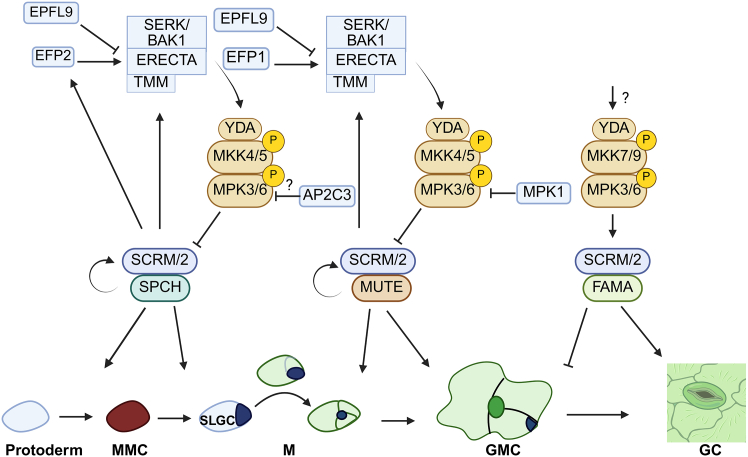
Schematic representation of the signal transduction pathway regulating stomatal development. Stomatal development follows a defined sequence of stages. A protodermal cell differentiates into a meristemoid mother cell (MMC), which undergoes asymmetric division to produce a meristemoid (M) and a stomatal lineage ground cell (SLGC). The meristemoid may divide further before transitioning into a guard mother cell (GMC), which divides symmetrically to form two guard cells (GCs) that comprise the stomatal pore. This process is regulated by a signaling cascade initiated by EPIDERMAL PATTERNING FACTORS (EPF1, EPF2, and EPFL9), which interact with ERECTA-family receptor kinases (ERECTA), the co-receptor SERK/BAK1, and the leucine-rich repeat receptor-like protein TOO MANY MOUTHS (TMM). These receptors activate a MAPK cascade involving YODA (YDA), MAPK kinases (MKK4/5 or MKK7/9), and MAPKs (MPK3/6), which differentially regulate three basic-helix-loop-helix (bHLH) transcription factors: SPEECHLESS (SPCH), MUTE, and FAMA. SPCH, in complex with SCREAM (SCRM)/SCRM2, promotes MMC formation and asymmetric divisions. MUTE, also functioning with SCRM/2, drives meristemoid differentiation into GMCs. FAMA, with SCRM/2, governs the final differentiation of guard cells. The MAPK cascade negatively regulates SPCH and MUTE to control stomatal entry and progression. AP2C3 acts as a negative regulator of MAPK signaling, likely by dephosphorylating MPK3/6, and is proposed to play a key role during the early MMC-to-meristemoid transition. AP2C3 also influences cytoskeletal dynamics, affecting spindle orientation through microtubule-associated proteins, thereby linking cell division mechanics with stomatal patterning. MPK1 may further modulate MUTE activity. Sharp arrows and blunt arrows indicate promotive and inhibitory effects, respectively. Created with www.Biorender.com.

AP2C3, a clade B PP2C, functions as a negative regulator of MPK3 and MPK6 during stomatal development, particularly within stomatal lineage cells (Umbrasaite et al., 2010) (Figure 3). Overexpression of AP2C3 induces excessive stomatal differentiation, resembling the phenotype observed in *mpk3/mpk6* mutants. Although *ap2c3* loss-of-function mutants do not display pronounced developmental defects—likely due to redundancy with other phosphatases—AP2C3 plays a role in regulating asymmetric cell divisions and microtubule dynamics (Umbrasaite et al., 2010). Specifically, AP2C3 interferes with phosphorylation of EB1c, a microtubule-associated protein involved in spindle orientation and chromosome segregation during progression through the stomatal lineage (Kohoutová et al., 2015). AP2C3 likely functions during the transition from meristemoid mother cell to meristemoid, a key step in establishing stomatal identity.

#### Stomatal movement in response to stress

**Figure 4:**
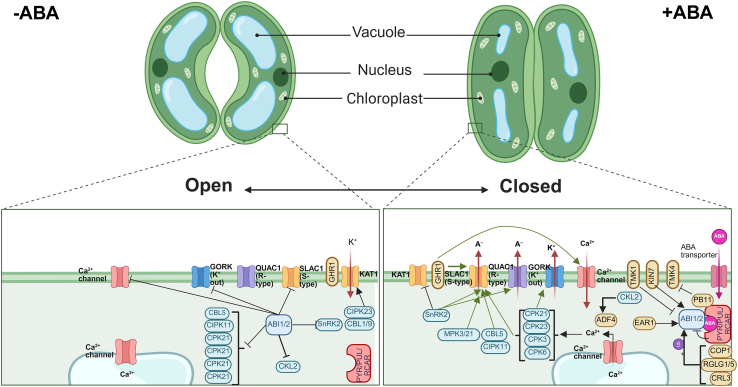
ABA-mediated regulation of stomatal movement under non-stress (−ABA) and stress (+ABA) conditions. Under non-stress conditions, guard cells maintain open stomata through active K^+^ uptake via inward-rectifying potassium channels (KAT1) and minimal Ca^2+^ influx, resulting in high turgor pressure. During this state, cytosolic Ca^2+^ levels remain low, and signaling components such as CALCINEURIN B-LIKE PROTEINs (CBLs), CBL-INTERACTING PROTEIN KINASEs (CIPKs), CALCIUM-DEPENDENT PROTEIN KINASEs (CPKs), and the phosphatase ABA-INSENSITIVE 1 (ABI1) remain inactive. CASEIN KINASE1-LIKE 2 (CKL2) also suppresses actin cytoskeleton remodeling by inactivating ACTIN-DEPOLYMERIZING FACTOR (ADF). Upon exposure to abiotic stress, elevated levels of abscisic acid (ABA) bind to PYRABACTIN RESISTANCE 1 (PYR1)/PYR1-LIKE (PYLs)/REGULATORY COMPONENTS OF ABA RECEPTORS (RCARs), leading to inactivation of ABI1/ABI2 and triggering the ABA signaling cascade. This activation results in increased cytosolic Ca^2+^ through Ca^2+^ channels, and the activation of CPKs (e.g., CPK6), CIPK23/CBL1.9 complexes, and SNF1-RELATED PROTEIN KINASE 2 (SnRK2). These kinases phosphorylate and activate downstream effectors such as the anion channel SLOW ANION CHANNEL-ASSOCIATED 1 (SLAC1) and GUARD CELL OUTWARD RECTIFYING K^+^ CHANNEL (GORK). Concurrently, MAPKs such as MPK3 and MPK21 are activated, contributing to the signaling network. The combined efflux of anions and K^+^, followed by water loss, reduces turgor pressure and induces stomatal closure. Additional regulatory factors include ENHANCER OF ABA RESPONSE 1 (EAR1), which enhances ABI1 phosphatase activity; PLANT U-BOX PROTEIN 11 (PUB11), which targets ABI1 for ubiquitin-mediated degradation; and RING DOMAIN LIGASEs 1 and 2 (RGLG1/2), which regulate protein turnover. CONSTITUTIVE PHOTOMORPHOGENIC 1 (COP1) may also contribute to signal integration during stress. This intricate network of kinases, phosphatases, ion channels, and secondary messengers coordinates ABA-induced stomatal closure to minimize water loss during abiotic stress. Sharp arrows and blunt arrows indicate promotive and inhibitory effects, respectively. Created with www.Biorender.com.

Clade A PP2Cs, particularly ABI1 and ABI2, play central roles in ABA-mediated stomatal closure (Figure 4). In the absence of ABA, these phosphatases inhibit activation of slow anion channel-associated 1 (SLAC1) (Lee et al., 2009). Upon ABA binding to PYR/PYL/RCARs, ABI1 and ABI2 are inhibited, allowing OST1 and calcium-dependent protein kinases to phosphorylate SLAC1 and quick anion channel 1, facilitating anion efflux and promoting stomatal closure (Brandt et al., 2015). ABI2, but not ABI1, also suppresses SLAC1 activation through GHR1, indicating distinct, non-redundant roles in guard cell signaling (Hua et al., 2012). Further molecular and biochemical studies are needed to clarify why ABI2, but not ABI1, inhibits GHR1-mediated SLAC1 activation and to elucidate the mechanisms underlying this differential regulation.

#### Stomatal movement in response to CO_2_ signaling

AP2C3 is uniquely involved in CO_2_-induced stomatal closure. This clade B PP2C contains an intrinsically disordered region within its N-terminal domain that enables direct CO_2_ sensing—a feature absent in other clade B members (Zhang et al., 2022). This disordered region is enriched in polar amino acids and includes essential serine and threonine residues required for CO_2_ responsiveness, although the downstream signaling pathway remains unclear. It is of particular interest to determine whether AP2C3 functions solely through PKMAPK-dependent dephosphorylation to mediate CO_2_-induced stomatal closure, or if phosphorylation-independent mechanisms also contribute to this response.

#### Stomatal movement in response to light

Clade D PP2Cs, including ADP6 and ADP9, contribute to light-regulated stomatal movement by dephosphorylating Thr^947^ of PM H^+^-ATPases (e.g., AHA1) in guard cells. This activity reduces ATPase function and promotes stomatal closure in darkness (Akiyama et al., 2022). Under blue light, the photoreceptors PHOT1 and PHOT2 phosphorylate Thr^947^ (Hayashi et al., 2011), reactivating H^+^-ATPases and thus promoting membrane hyperpolarization, K^+^ influx, and stomatal opening (Jezek and Blatt, 2017). Intriguingly, *adp6/adp9* double mutants do not fully abolish H^+^-ATPase dephosphorylation, suggesting compensation by other clade D PP2Cs or phosphatases with redundant functions. It remains unclear whether these additional phosphatases directly target PM H^+^-ATPases. Moreover, the mechanisms by which blue light regulates PP2CD activity are not fully understood. Because Thr^947^ phosphorylation can occur in the absence of blue light (Akiyama et al., 2022), it is possible that blue light primarily acts by inhibiting the dephosphorylation activity of PP2CDs involved in stomatal regulation. Future research should aim to elucidate how blue light influences PP2CD activity at the molecular level.

### Roles of PP2Cs in root growth and nutrient uptake

#### ABA-mediated root development

Clade A PP2Cs are central to the ABA-dependent regulation of root growth under stress conditions. In *Zea mays*, ZmPP2C-A10 enhances drought tolerance by fine-tuning ABA signaling to promote root elongation during water scarcity (He et al., 2019). A similar function has been reported for the rice homolog OsPP108, which, when overexpressed in *A. thaliana*, improves root development under osmotic stress. These findings highlight a conserved role for clade A PP2Cs in integrating environmental signals into root developmental programs (Singh et al., 2015). Recent work has identified a mechanism linking ABA-activated SnRK2 kinases to sugar accumulation in roots during drought (Chen et al., 2022). SnRK2s phosphorylate SWEET sucrose transporters, facilitating sucrose accumulation in root tissues and enhancing root biomass and growth, thereby improving drought resilience. However, the identity of the PP2C(s) that regulate SnRK2 activity in this pathway remains unknown, revealing a critical gap in understanding how PP2Cs connect ABA signaling with carbohydrate allocation in the root system.

#### Roles of PP2Cs in K^+^ regulation

Clade A PP2Cs also modulate K^+^ channel activity, which is vital for nutrient uptake and ion homeostasis in roots. AHG3 regulates several K^+^ transporters, including AKT2 and AKT3—channels important for phloem K^+^ circulation—and GORK, a K^+^ efflux channel in guard cells (Chérel et al., 2002; Lefoulon et al., 2016). AHG3 also inhibits CALCINEURIN B-LIKE PROTEINS–INTERACTING PROTEIN KINASE 6 (CIPK6), a kinase that activates AKT1, the primary K^+^ uptake channel in root epidermal cells under low-K^+^ conditions (Wang and Wu, 2010; Lan et al., 2011). HAI2, another clade A PP2C, negatively regulates AKT1 by dephosphorylating CIPK23, suggesting the existence of parallel regulatory modules converging on the same transporter (Lan et al., 2011). The spatial and temporal distinction between AHG3- and HAI2-mediated regulation of K^+^ signaling remains an open question.

Clade B PP2C AP2C1 contributes to K^+^ homeostasis through a different mechanism. AP2C1 interacts with CIPK9 but does not regulate AKT1 activity. Instead, this signaling module influences the expression of *HAK5*, a high-affinity K^+^ transporter induced under K^+^ deficiency (Singh et al., 2018). In *ap2c1* mutants, *HAK5* expression is strongly upregulated, whereas it is reduced in *cipk9* mutants, indicating that the AP2C1–CIPK9 module controls K^+^ uptake at the transcriptional level (Singh et al., 2018). These findings raise important questions about how plants selectively recruit different PP2C clades in response to K^+^ deficiency. One plausible explanation is spatial compartmentalization within root tissues, whereby AP2C1, AHG3, and HAI2 function in distinct microdomains to mediate local versus systemic responses.

### Roles of PP2Cs in other developmental processes

#### Meristem maintenance and developmental signaling

Clade C PP2Cs were the first to be implicated in shoot meristem establishment and maintenance (Yu et al., 2000). Among them, POLTERGEIST (POL) and POL-like 1 (PLL1) are key components of the CLAVATA (CLV) signaling pathway, which coordinates the balance between stem cell identity and differentiation in the shoot apical meristem (SAM). Loss-of-function mutations in *POL* and *PLL1* suppress the overproliferation phenotype of *clv* mutants but also lead to SAM termination, indicating that POL and PLL1 function downstream of CLV3 as negative regulators of stem cell proliferation (Yu et al., 2003; Song and Clark, 2005). At the molecular level, POL and PLL1 are serine/threonine phosphatases that interact with receptor complexes to regulate signal transduction. Recent studies have shown that both proteins are phosphorylated by receptor-like cytoplasmic kinases (RLCKs), such as PBL34, at conserved S-X-X-L motifs in their N-terminal domains (DeFalco et al., 2022). Phosphorylation at these sites induces dissociation of POL and PLL1 from receptor complexes, thereby modulating downstream signaling pathways. Although PLL4 and PLL5 share this phosphorylation-dependent regulatory mechanism, only POL and PLL1 are directly involved in SAM maintenance. These characteristics raise important questions about RLCK–PP2C specificity: How do distinct RLCKs selectively recognize and phosphorylate particular PP2C targets? Do sequence variations within the S-X-X-L motifs of POL- and PLL-type PP2Cs confer different affinities for specific kinases, and how might this structural specificity contribute to their functional divergence in developmental regulation?

#### Regulation of developmental timing

Clade E PP2Cs have also been linked to growth and developmental regulation under non-stress conditions. In *A. thaliana*, EGR1 (AtPP2CF1) promotes biomass accumulation by enhancing inflorescence stem elongation, suggesting a role in cell expansion and vascular development (Sugimoto et al., 2014). Similarly, in *Brassica juncea*, BjuPP2C52, a functional homolog of AtPP2CF1, influences flowering time, indicating conserved roles for clade E PP2Cs in developmental phase transitions (Zeng et al., 2022). Although the interacting partners of these clade E PP2Cs remain unidentified, it is plausible that they regulate development by dephosphorylating transcription factors or protein kinases involved in circadian rhythms and photoperiodic signaling. Their involvement in flowering time control suggests that they fine-tune developmental timing by integrating environmental cues with endogenous hormonal signals to modulate growth rates and phase transitions.

### Roles of PP2Cs in abiotic stress responses

#### Drought stress

Drought stress triggers the accumulation of ABA, which activates the canonical ABA–PYL–PP2C–SnRK2 signaling cascade. As previously described, clade A PP2Cs act as negative regulators of drought tolerance by dephosphorylating SnRK2s and maintaining ABA signaling in an inactive state (Jossier et al., 2009). However, PP2Cs from other clades also contribute to drought responses through distinct mechanisms. In *Z. mays*, the clade B phosphatase ZmPP84 impairs stomatal closure by dephosphorylating ZmMEK1, which prevents activation of ZmSIMK1 and disrupts ABA signal transduction (Guo et al., 2023). Clade E members EGR1 and EGR2 suppress root meristem activity under drought conditions by dephosphorylating microtubule-associated stress protein 1, thus restricting cell division (Longkumer et al., 2022). PIA1, a clade F PP2C, acts as a negative regulator of drought tolerance by repressing ABA-responsive gene expression (Huang et al., 2023b). In *T. aestivum*, the clade I phosphatase TaPP2C158 dephosphorylates TaSnRK1.1 and weakens the TaSnRK1.1–TaAREB3 transcriptional cascade, attenuating drought signaling (Wang et al., 2023b). This pathway functions independently of ABA receptors, representing a noncanonical, ABA-modulated mechanism distinct from the classical PYL–PP2C–SnRK2 module. Taken together, these findings highlight the clade-specific specialization of PP2Cs in drought responses. Further investigation into the spatial and temporal dynamics of PP2C–protein interactions across tissues may clarify how plants prioritize and coordinate drought adaptation; such efforts may help to identify novel targets for enhancing stress resilience beyond the canonical ABA pathway.

#### Extreme temperature stress

Responses to extreme temperatures involve extensive transcriptional reprogramming, membrane stabilization, and hormone signaling—processes in which PP2Cs exert regulatory control through dephosphorylation of signaling proteins and modulation of stress-responsive pathways. Under cold stress, AHG3 expression increases in guard cells, and its overexpression induces freezing sensitivity (Tähtiharju and Palva, 2001). ABI1 also acts as a negative regulator by suppressing OST1 activity, thus attenuating ABA signaling during cold exposure (Ding et al., 2015). Additionally, EGR2 inhibits OST1 independently of ABA, contributing to cold tolerance through an alternative regulatory route (Ding et al., 2019). These findings suggest that EGRs and ABI1/2 function either in parallel or in coordination to modulate OST1 activity, potentially within distinct subcellular compartments (e.g., EGRs at or near the plasma membrane and ABI1/2 in the cytosol or nucleus). In the context of heat stress, the clade D PP2C FORGETTER2 (FGT2) plays a role in heat stress memory (Urrea Castellanos et al., 2020). FGT2 interacts with phospholipase Dα2, an enzyme involved in generating phosphatidic acid, a lipid secondary messenger that contributes to the maintenance of heat-induced transcriptional memory. Whether FGT2 regulates heat–phospholipase Dα2 responses by modifying membrane lipids or by participating in phosphatidic acid–dependent signaling remains to be determined and warrants further investigation.

#### Salinity stress

Salt stress induces both osmotic and ionic challenges that are mitigated by PP2Cs through the regulation of ion transport and stress-responsive signaling pathways. ABI2 contributes to salinity adaptation by modulating the salt overly sensitive (SOS) pathway. It functions through CPK12-mediated activation of SOS2, which phosphorylates and activates the Na^+^/H^+^ antiporter SOS1, thereby promoting ion homeostasis (Ohta et al., 2003; Zhao et al., 2011). ABI2 also dephosphorylates the calcineurin CBL1–CIPK23 complex, a key regulator of the nitrate transporter NPF6.3, further influencing ionic balance under saline conditions (Léran et al., 2015). Other PP2C clades also regulate salt stress responses. Clade E phosphatases act as negative regulators by dephosphorylating GCN5, a histone acetyltransferase that activates stress-responsive genes, suppressing transcriptional responses essential for salt tolerance (Servet et al., 2008; Zheng et al., 2019). Within clade G, functional divergence is evident: AtPP2CG1 enhances salt tolerance via ABA-dependent upregulation of *RD29A/B*, *DREB2A*, and *KIN1* (Liu et al., 2012), whereas PP2C49 suppresses salt adaptation by downregulating HKT1;1-mediated Na^+^ retrieval (Chu et al., 2021). Silencing PP2C49 increases Na^+^ retention in roots, shielding shoot tissues from ionic toxicity. The clade F member TaPP2C1 improves salt tolerance in *Nicotiana benthamiana*, doubling plant survival under saline conditions, although its molecular targets remain unidentified (Hu et al., 2015). These findings underscore the complexity of PP2C-mediated salt stress signaling, characterized by clade specialization and cross-pathway integration. Future work should define the site- and stage-specific activity of PP2Cs and assess potential redundancy or synergy across clades during salinity adaptation.

### Roles of PP2Cs in biotic stress responses

**Figure 5:**
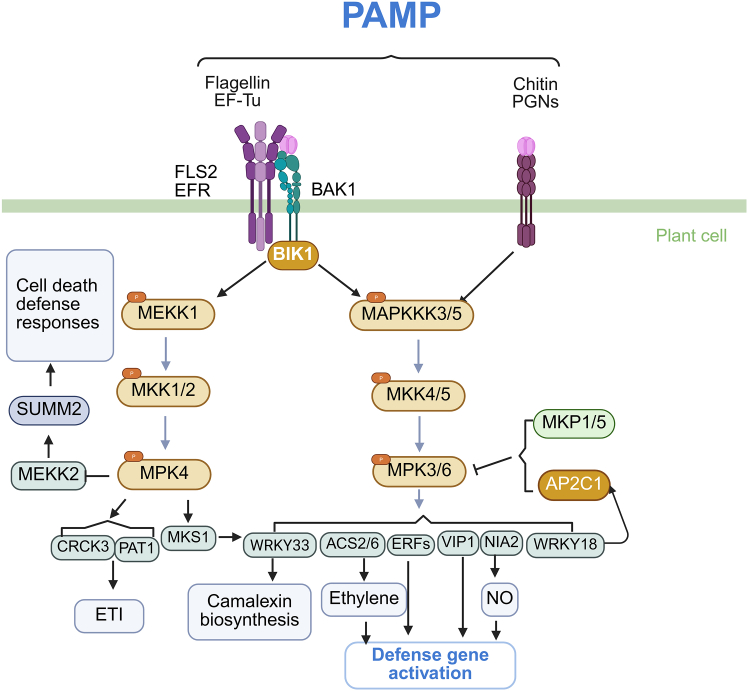
PAMP-triggered MAPK signaling cascades in plant innate immunity mediated by AP2C1. Recognition of bacterial flagellin (e.g., flg22) and elongation factor Tu (EF-Tu) is mediated by the pattern recognition receptors FLAGELLIN-SENSITIVE 2 (FLS2) and EF-TU RECEPTOR (EFR), which form complexes with the co-receptor BRASSINOSTEROID INSENSITIVE 1-ASSOCIATED KINASE 1 (BAK1). These receptor complexes activate BOTRYTIS-INDUCED KINASE 1 (BIK1), which transduces the signal to downstream mitogen-activated protein kinase (MAPK) modules. One signaling branch activates MAPK kinase kinase 1 (MEKK1), which phosphorylates MKK1 and MKK2, leading to the activation of MPK4. MPK4 regulates defense responses by modulating downstream effectors such as CALMODULIN-BINDING RECEPTOR-LIKE CYTOPLASMIC KINASE 3 (CRCK3) and PLEIOTROPIC ARABIDOPSIS TOXIN 1 (PAT1), contributing to effector-triggered immunity (ETI), and suppresses cell death through the SUMM2–MEKK2 pathway. A parallel MAPK cascade involves activation of MAPKKK3 and MAPKKK5, which phosphorylate MKK4 and MKK5, subsequently activating MPK3 and MPK6. These kinases phosphorylate a range of transcriptional regulators, including WRKY33, ETHYLENE RESPONSE FACTORs (ERFs), VIRE2-INTERACTING PROTEIN 1 (VIP1), and NITRATE REDUCTASE 2 (NIA2), leading to activation of genes involved in camalexin biosynthesis, ethylene production (via ACS2/6), and nitric oxide (NO) signaling. WRKY18 also contributes to transcriptional activation of defense genes. MAPK activity is negatively regulated by MAPK PHOSPHATASES 1 and 5 (MKP1 and MKP5) and by the clade B phosphatase AP2C1, which dephosphorylate MPK3 and MPK6 to prevent excessive immune responses. In addition to bacterial PAMPs, fungal and bacterial cell wall components such as chitin and peptidoglycan (PGN) also pathogen-associated molecular pattern (PAMPs)activate similar MAPK signaling cascades. Sharp arrows and blunt arrows indicate promotive and inhibitory effects, respectively. Created with www.Biorender.com.

PP2Cs play key roles in plant immunity by modulating MAPK cascades, hormone signaling pathways, and redox-sensitive networks. Clade B phosphatases, such as AP2C1, deactivate MAPKs—including MPK3, MPK4, and MPK6—via a conserved kinase interaction motif, preventing overactivation of immune signaling (Meskiene et al., 1998; Schweighofer et al., 2007; Ayatollahi et al., 2022) (Figure 5). AP2C1 is strongly induced upon recognition of pathogen-associated molecular patterns, where it suppresses ethylene biosynthesis and downstream defense gene expression, functioning as a negative regulator of early immune responses. Loss-of-function *ap2c1 mutants* exhibit enhanced resistance to *Pseudomonas syringae* pv. *tomato* DC3000 and *Fusarium oxysporum*. Double mutants lacking both *AP2C1* and *MKP1* display constitutive immune activation, characterized by sustained MAPK activity, elevated levels of salicylic acid (SA), ethylene, and camalexin, and an autoimmune-like phenotype (Shubchynskyy et al., 2017; Guo et al., 2022).

Clade A PP2Cs exhibit distinct regulatory roles in biotic stress responses. For example, ABI1, but not ABI2, enhances resistance to *Leptosphaeria maculans* by modulating expression of the *Resistance to Leptosphaeria maculans 1* gene (Staal et al., 2006; Kaliff et al., 2007). ABI2 also participates in redox-mediated immune signaling. Upon pathogen-induced oxidative bursts, ABI2 forms a disulfide bridge with the peroxiredoxin PRXIIB, leading to its inactivation and promoting stomatal closure (Bi et al., 2022). Despite the close homology between ABI1 and ABI2 and their overlapping roles in ABA signaling, the molecular basis for their selective regulation of biotic resistance remains unclear. Structural differences may underlie the specificity of ABI1 in disease resistance, but further investigation is needed to test this hypothesis. The redox-sensitive regulation described above also highlights the potential involvement of PP2Cs in thiol-based immune signaling and raises the broader question of whether other PP2Cs engage in redox-dependent control of immunity.

HAI-type clade A phosphatases suppress MPK3 and MPK6 during pathogen infection, a function that is antagonized by the bacterial toxin coronatine through activation of jasmonate signaling via MYC2 (Mine et al., 2017). This suppression is reversed by the long non-coding RNA FL7, which binds and inhibits HAI1, restoring MAPK activity and immune responses (Ai et al., 2022). This finding introduces a novel layer of post-transcriptional immune regulation involving clade A PP2Cs. MPK3 and MPK6 are also regulated by clade B PP2Cs, suggesting overlapping phosphatase activity across clades. This regulation raises important questions regarding substrate specificity: How do PP2Cs from different clades selectively target common MAPKs during biotic stress? Is there functional redundancy or a dynamic handoff mechanism between clades depending on the nature or stage of the immune response? Furthermore, does crosstalk between clade A and clade B PP2Cs enable coordination between immune signaling and other physiological pathways, such as growth regulation and metabolism? Addressing these questions will be essential for understanding how PP2Cs integrate immune responses into broader stress adaptation frameworks.

Clade E PP2Cs, such as Pic14 in *Solanum lycopersicum*, negatively regulate Pto/Prf-mediated immunity by modulating MAPK signaling (Qiu et al., 2022; Chakraborty, 2024). Whether other clade E members similarly influence discrete immune responses through MAPK interactions remains unclear. Notably, as discussed earlier, clades A and B also modulate plant immunity by acting on components upstream or downstream of MAPK cascades. Despite structural differences, their shared targeting of MAPKs suggests a potential case of functional convergence. This convergence likely reflects independent evolutionary adaptations across clades to regulate MAPK signaling under distinct environmental and physiological conditions, rather than redundancy. Further research is needed to determine whether additional shared interactors contribute to this convergence, which could illuminate the molecular logic underlying PP2C diversification within plant stress signaling networks.

Clade F phosphatases also participate in immune regulation against bacterial pathogens. WIN2 (homologous to At4g31750) enhances resistance to bacterial infection (Lee et al., 2008), whereas PAPP2C suppresses SA-dependent hypersensitive responses through its interaction with the RPW8.2 resistance protein (Wang et al., 2012). PIA1 integrates SA and jasmonate signaling during effector-triggered immunity (Widjaja et al., 2010). However, it remains unclear whether clade F PP2Cs function exclusively in response to bacterial pathogens or if this apparent specificity reflects broader, yet uncharacterized, roles in defense against other pathogen types.

Collectively, PP2Cs from multiple clades contribute to plant immunity by coordinating dephosphorylation of immune components, regulating hormone crosstalk, and integrating spatially resolved signaling. These functions ensure finely tuned, context-specific defense responses that balance immune activation with growth and metabolic demands.

## Concluding remarks and future perspectives

Although the regulatory roles of clade A and clade B PP2Cs are well established, the mechanistic functions of less-characterized clades, such as PP2CG and PP2CI, remain largely unexplored. Moreover, functional diversity exists even within individual clades, with members exerting distinct effects on stress responses. This indicates that clade classification alone is insufficient to predict specific regulatory functions. Systematic functional characterization of individual PP2Cs is needed, with emphasis on identifying their interacting partners, subcellular localization, and temporally resolved expression patterns under defined stress and developmental conditions. Most current PP2C research is concentrated in model species, leaving a substantial gap in our understanding of their roles in non-model or agronomically important plants. Investigating PP2C functions in diverse plant lineages, particularly those lacking specific isoforms, may uncover lineage-specific adaptations and help define core regulatory mechanisms conserved throughout plant evolution.

Current PP2C research has primarily focused on leaves and roots. Extending these investigations to reproductive tissues at single-cell resolution may uncover cell-type-specific functions of PP2Cs in processes such as gametogenesis, fertilization, and early seed development—areas that remain largely unexplored. Some PP2Cs have been associated with the hypersensitive response, a localized form of programmed cell death (PCD), suggesting potential roles in immune-related cell death. However, direct evidence of PP2C involvement in core PCD signaling remains limited. Although PP2Cs are known regulators of reactive oxygen species production, it is unclear whether they function upstream of reactive oxygen species-mediated PCD.

Beyond stress signaling, the roles of PP2Cs in developmental and symbiotic processes are poorly understood. The absence of data on PP2C activity at beneficial plant–microbe interfaces represents a critical knowledge gap. For example, it remains unknown whether specific PP2Cs modulate hormonal gradients in meristematic zones or contribute to establishing symbiotic compatibility. Targeted studies are needed to determine whether PP2Cs function as facilitators of microbial accommodation or suppressors of immune responses during symbiosis. In summary, despite substantial progress in understanding certain PP2C clades, many members remain functionally uncharacterized, especially in developmental and non-model systems. Elucidating their roles within specific signaling pathways is essential for a comprehensive understanding of PP2C function in plant physiology. Advancing this knowledge will provide opportunities to manipulate PP2C activity for crop improvement and enhance stress resilience in sustainable agricultural systems.

## Funding

Financial support for this work was provided by the 10.13039/501100001809National Natural Science Foundation of China (grants U22A20495 and 32072588).

## Acknowledgments

The authors apologize to those colleagues whose work could not be cited due to word and reference limitations. We would like to thank the three anonymous reviewers for their insightful and constructive comments, which significantly improved this manuscript. We also appreciate the support from Northeast Agricultural University and the China Scholarship for Foreign Students granted to Zainab Qamer. No conflict of interest declared.

## Author contributions

H.G., A.W., and Y.Z. designed the content and structure of the review; H.G. and Z.Q. wrote the first draft of the manuscript; and A.W. edited the manuscript. All authors read and approved the final version of the manuscript.
